# The Divorcement of Dental Literature from Trade

**Published:** 1898-07

**Authors:** 


### Editorial.

### THE DIVORCEMENT OF DENTAL LITERATURE FROM TRADE.

Our pages this month are largely devoted to a consideration of this subject. There should be no controversy with the views entertained by writers and speakers in advocacy of a more thoroughly professional spirit. The time is ripe for a readjustment of professional ideas and the relations of dentistry with trade, and there can be no two opinions in regard to the value of the papers and discussions presented.

It will probably be said, by way of criticism, Why repeat the old story? The time has not come when dentistry can be divorced from trade. This is the question conservatism always propounds when an effort is made to effect an alteration in the existing order. The active thinker who desires change, not for the mere pleasure of innovation, but from an earnest desire to improve on past methods, will not rest quiet because apparently insuperable difficulties lie in the path.

The history of dentistry is replete with the work of individuals. The very best examples of professional spirit are to be found here, and it is safe to assert that it will be difficult to find elsewhere more instances of devotedness and self-sacrifice in the development of a profession than has been manifested in dentistry. That this has not been universal is to be lamented, but the conditions were not present in the past to warrant an extended devotion to professional ethics.

The time was, and not far distant in the past, when it required all the patience and mental strength of the men in dentistry to deal with the problems daily presenting in practice. Very few, it is presumed, as they follow the prescribed rules for the preparation of an artificial denture, think of the great labor and constant experimentation required to reach the results accepted to-day. Only those who have followed the changes step by step can appreciate this, and it is equally applicable to every operation in prosthetic dentistry as well as other branches. While dentistry began more than a thousand years in the past history of the world, it had no development worthy the name prior to the middle of the present century.

This burden of growth was carried by every worker of intelligence, and in the effort to improve practical methods the cultivation of the professional spirit was lost to view, and a species of paternalism was established in the minds of dentists, producing a weakness in them as individuals and destructive to the vigor of the entire professional body.

The weak point in dentistry was that in a too extended cultivation of the practical the intellectual became submerged. The industrious student of the past literature of dentistry will find little to compensate him for his labor, it being in fact almost a barren field, with here and there a cultivated spot intensifying the surrounding desolation.

The world during its dark period is indebted to the monasteries of Europe for the partial preservation of its literature, and dentistry should give credit to the trade periodicals for the preservation of the most active portion of its history during the last half of the present century. What a treasure it would have been could we have had placed upon our library shelves the history, month by month, of the early period of the same century and of the last. We have only the record of individual experience in the few books issued.

It must not be forgotten, therefore, that we owe a debt of gratitude to the trade interest in thus preserving the most valuable facts at a time when the active workers were unable or unwilling to make any move for the intellectual advancement of dentistry. The fact of this indifference is plainly manifested in the failure of the first professional journal. Its life was short, and its pages do not give marked evidence of intellectual force broadcast in the dental field. It was evident that it was left to the few to labor untiringly and without reward save that which belongs to a duty unselfishly performed.

The so-called trade journals have therefore given a service that must be gratefully recognized and remembered, and they are still performing it with varying degrees of success. It is understood that this service was not unmingled with the selfishness of trade, but that it has helped, in no minor degree, to build the foundations upon which the superstructure will rest for the future, remains a fact in dental history.

The question that now concerns the dental profession is, Has it reached a point in its development when the crutches, on which it has leaned for support in the past, may be cast aside? The answer to this is given in the articles published. It is needless to say that this journal is the exponent of the sentiments there detailed, sentiments which recognize that dentistry has arrived at a stage when old methods must be gradually discarded and a periodical literature established exclusively its own. That this cannot be an easy task is recognized both theoretically and experimentally, but the true life of the profession can never be exemplified in any other way and the progress of the age and of our work demands the change. This means no disparagement of the ability with which some of the journals are conducted and issued by supply houses. It is acknowledged that they are ably edited by men active as leaders in the intellectual advancement of dentistry. The fact that they are thus lending their aid in this direction serves to enforce the importance of a change in the prevailing sentiment of the profession. Instead of dividing their interests their intellectual powers might be better devoted to the upbuilding of the forces that make for progress in their calling. The important aid given by trade to dentistry has been fully set forth, not only in this article, but in the leading thoughts of previous issues, credit having been ungrudgingly given whenever or wherever due, but while this remains as written there is still a very large proportion of selfishness that would seem to cling to all connected therewith.

The argument set forth by one of the editors at the meeting alluded to, which seems to the writer to be unworthy the origin, in that they appear to glorify the ability of the trade journals to crush all attempts on the part of the profession to introduce an independent literature. It is the old story of the power of money. The quotation from the remarks of the editor indicates a spirit not commendable or worthy of imitation. He says, "Do the members of the Institute think they can start a wave over the country which will support a dental magazine and reach the standard which has been set by some of the so-called trade journals, when it is a certain fact that it will be reached only by a loss of from ten to eighteen thousand dollars per annum?"

It is understood that the publication of a journal is an expensive business, and that the trade houses are willing to spend large sums from which they can expect no direct returns for the privilege of bringing their wares to the attention of the profession. In doing this they simply cater, not to professional, but to selfish interests, and the glorification of the fact, if such it be, that they are able, through the aid of capital, to do more than can be done by an unselfish work for the good of others, does not sound well coming from the source from which it emanated.

It is acknowledged that the dental profession has had to contend with the power of money. This has debauched it in the past, and will continue this work, until men can be found willing to make sacrifices equal to that made by this power and in the same direction.

While the "paltry salaries" dwelt upon may not be the inducement which has led men to lend their energies to the development of trade, it nevertheless remains a fact that, were these services not given, trade journalism would wither in spite of capital.

More than this, individuals and societies are engaged, with a few honorable exceptions, in feeding the pages of the trade journal under the specious plea that capital can illustrate gratuitously and that their articles will be given a larger circulation. The first claim is not correct, for a professional journal can and does illustrate, and the second is equally erroneous, for no article worthy to live is ever buried in any publication. This is proved by the extensive repetition, in both domestic and foreign journals, of all original matter. This criticism is made generally by writers whose articles are produced, not through original researches, but from ideas gathered hither and thither, frequently without due credit. These, of course, have not an extended circulation, and probably have but few readers no matter where published.

It is unprofessional, in the opinion of the writer, for a society to barter its work to the highest bidder. It is here that capital always has the advantage, not to its discredit, but to the society that takes advantage of the offers made. The example has been set by the leading association of the country, and has been followed by subordinate societies until we have lost, in large degree, professional instincts, and have descended to the region where the sale of our best thoughts has become a settled practice.

That all this is wrong and unprofessional needs no argument. It is even admitted by those engaged in the practice, with the accompanying excuse: "That the money is needed for other purposes, and it is therefore right and a good business operation to sell proceedings to the best bidder procurable."

The hour is upon us in which the dental profession must stand for higher things. While it is not supposed there will be any marked or sudden change for the better, there should be an immediate and sharp dividing line drawn between that which has sustained the profession in the past and that which will add to its honor and effectiveness in the future. Dentistry needs, and must have, a periodical literature all its own, and the time has come when this must be fully recognized. The intelligence combined in its ranks must work for it until capital is forced to seek other means to satisfy the demand for a knowledge of its wares or disclaim the present attitude of two-thirds trade and one-third professional, for one wholly of trade with no abortive attempt to guide dental thought into channels not recognized as strictly ethical. In this way both branches of the great work of building up dentistry will be developed with self-respecting labor upon both sides and with less of the crimination and recrimination now so unpleasantly apparent, resulting in injury to both wings of an honorable calling.

